# Comprehensive evaluation of photoelectrochemical performance dependence on geometric features of ZnO nanorod electrodes†

**DOI:** 10.1039/d3na00089c

**Published:** 2023-05-24

**Authors:** Ali Can Guler, Jan Antos, Milan Masar, Michal Urbanek, Michal Machovsky, Ivo Kuritka

**Affiliations:** a Centre of Polymer Systems, Tomas Bata University in Zlin Tr. T. Bati 5678 760 01 Zlin Czech Republic kuritka@utb.cz; b Department of Chemistry, Faculty of Technology, Tomas Bata University in Zlín Vavrečkova 5669 760 01 Zlín Czech Republic

## Abstract

The impact of geometric features, light absorption spectra, and electrochemical active surface area on photoelectrochemical properties was investigated in this work. Nanoforests of ZnO nanorods with rationally controlled morphologies were grown on ITO substrates by the hydrothermal method and utilized as a model for this purpose. The size of the nanorods was systematically adjusted by varying the concentration of polyethyleneimine as a cation surfactant in the growth solution. It was found that the emergent geometric characteristics (*i.e.* the aspect ratio) increased almost at the same pace as the electrochemically active surface area, but the light scattering effect slightly increased as a result of the random spatial orientation of the nanorods. The large surface area and the void space between nanorods increased the photon-to-current conversion efficiency by promoting the hole transfer process at the electrode/electrolyte interface. A maximum photocurrent density of 0.06 mA cm^−2^ (0.5 V *vs.* NHE) for smaller diameter and length ZnO nanorods (ZnO–P1) was obtained under 365 nm UV light illumination. Additionally, we provide visual evidence that a shorter photogenerated hole diffusion distance results in improved charge separation efficiency using Mn^2+^ as the photogenerated hole imaging agent. Therefore, the present work demonstrates a facile strategy for nanoforest morphology improvement for generating strong contact at the ZnO NR electrode/electrolyte interface, which is favourable in energy conversion and storage technologies.

## Abstract 2

Polyethyleneimine use in the synthesis of ZnO changed the aspect ratio and void space of ZnO NRs, with the ∼40 nm NR diameter showing the highest ECSA and thus PEC properties. Charge transfer and transport were facilitated due to unique geometrical features.

## Graphical abstract

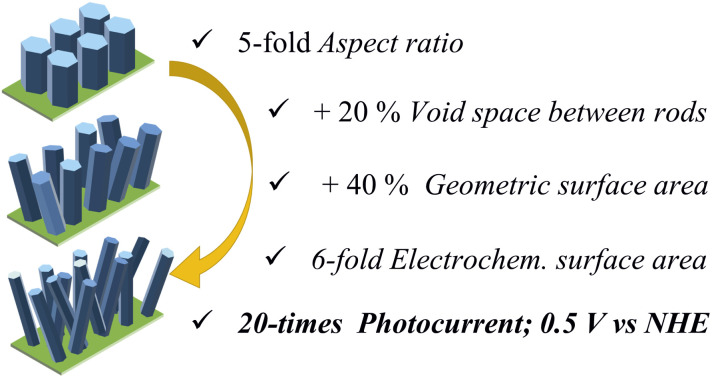

## 1. Introduction

Photoelectrochemical (PEC) cells powered by solar energy are widely utilized in various applications, *i.e.* electrochemical photovoltaic cells, dye sensitized solar cells and light emitting cells besides degradation of toxic organic pollutants and hydrogen fuel generation.^1,2^ However, low photoconversion efficiency, sustainability and high production costs are obstacles to the commercialization of PEC technology. The overall cost and performance of a PEC device are substantially determined by the properties of the semiconductor materials used as photoelectrodes. In fabricating photoanodes, metal oxide semiconductors have special importance owing to their low cost, stability under harsh aqueous conditions and resistance to photocorrosion. Among the most thoroughly explored metal oxides, zinc oxide (ZnO) is a promising solar energy material with controllable intrinsic n-type conductivity and a wide direct band gap (*E*_g_ = 3.37 eV).^3^ ZnO exhibits more outstanding electrical properties than its counterparts. For example, the measured mobility of a single ZnO nanowire in solar cells is 1–5 cm^2^ V^−1^ s^−1^, and accordingly the estimated electron diffusivity is 0.05–0.5 cm^2^ s^−1^. This value is several hundred times greater compared to the highest reported diffusivity for TiO_2_.^4^ Additionally, the conduction band minimum and valence band maximum of ZnO lie at −0.31 V *vs.* the normal hydrogen electrode (NHE) and 2.89 V *vs.* NHE, respectively, making it convenient for the water oxidation reaction (1.23 V *vs.* NHE).^5^

ZnO possesses diverse nanostructures, whose configurations are more versatile than those of any existing nanomaterials including carbon nanotubes.^6^ In comparison with bulk materials, one-dimensional (1D) nanostructures such as nanorods (NRs) offer several benefits in terms of charge dynamics. In fact, the diameters of the radial dimensions of the NRs are at or below the characteristic length of many fundamental solid state phenomena: the exciton Bohr radius, exciton diffusion length, wavelength of light, phonon mean free path, *etc.* The physical properties of the semiconductor are henceforth altered on the confined nanorod surfaces.^7^ Besides this, fewer electron trapping sites formed in single-crystalline NRs enhance electron transfer. However, since the specific surface area of NRs is not high enough, it limits the light absorption ability and the charge transfer process.^8,9^ For this reason, fabricating single crystal 1D ZnO NRs with a high specific surface area can greatly advance the efficiency of PEC cells.

Many reports have examined the PEC performance of ZnO NRs depending on the change in their morphology (size and shape).^10,11^ Recently, Govatsi *et al.* prepared ZnO nanowires with different average diameters in the range of 40–260 nm to investigate the role of morphology and the polar surfaces in PEC performance.^12^ A maximum applied bias photoconversion efficiency of ∼ 6.3% for nanowires with a 120 nm average diameter was linked to the increased specific surface area. Furthermore, the effect of diameter change on ZnO NRs in photoelectrochemical water splitting was investigated by Babu *et al.*, and the highest photoconversion efficiency was obtained from nanorods with the smallest diameter (45 nm).^13^

One possible way to prepare ZnO NRs with longer lengths but smaller diameters is by using capping agents. Joo *et al.* observed more than 3 orders of magnitude advancement in the aspect ratio for ZnO NRs modified by the face selective electrostatic interaction of non-zinc complexes.^14^ On synthesizing ZnO NRs, polyethyleneimine (PEI) can decrease the rod diameter and increase the rod length by selective absorption to lateral crystal facets.^15^ Recently, Sun *et al.* reported the synergistic influence of PEI and ammonia on the nanoarchitecture of ZnO nanoforests for PEC water splitting.^16^ In this comparative study, willow-like ZnO nanoforests stood out with their maximum current density of 0.919 mA cm^−2^ at 1.2 V (*vs.* Ag/AgCl) as charge migration is improved in longer branches. All these studies propound that the geometric features of ZnO NRs have critical importance in light absorption and charge transfer kinetics, thus in the PEC water splitting application. However, the relationship between the nanoarchitecture and the photoelectrochemical characteristics is still not completely understood.

In this work we present a detailed study of the influence of ZnO nanorod morphology on its photoelectrochemical properties. Thus, three different ZnO NRs with controlled diameters ranging from 45 nm to 115 nm were grown on ITO substrates *via* hydrothermal synthesis under different PEI concentrations. This sample series was used as a model to explore the effect of their geometry on light absorption and electrochemical active surface area. ZnO NRs with the smallest diameter (prepared with the highest PEI concentration) manifested a promising geometry for light harvesting and PEC applications. Moreover, we provide visual evidence for hole transport *via* Mn^2+^ as a hole imaging agent. This work provides a better insight into understanding charge transport in nanostructured photoelectrodes and the interrelationship between surface geometry and photoelectrochemical performance.

## 2. Experimental

### 2.1 Preparation of the seed layer

Indium tin oxide coated glass substrates (ITO, 5–15 Ω sq^−1^, Sigma Aldrich) were cut into square pieces of approximately 1 × 1 cm. These substrates were ultrasonically cleaned in a mixed solution of alkaline concentrate (Hellmanex III) and deionized water, isopropanol, and acetone and dried in an air atmosphere. A UV-ozone cleaner was also used to remove the remaining contaminants. A ZnO seed layer was prepared by the sol–gel spin coating technique followed by the calcination process. Zinc acetate dihydrate (Zn(CH_3_CO_2_)_2_·2H_2_O, 99.0%, Penta) and diethanolamine ((CH_2_CH_2_OH)_2_NH, 99.0%, CDH Fine Chemicals) were dissolved in isopropanol at room temperature. The diethanolamine to zinc acetate dihydrate molar ratio was retained at 1 and the concentration of zinc acetate was 0.8 M. The mixture was stirred at 50 °C for an hour and aged overnight. The resulting colloidal solution was spin-coated onto the cleaned substrates at 3000 rpm for 30 s. The ZnO NP seed coated substrates were crystallized in an air atmosphere at 400 °C for an hour.

### 2.2 Growth of ZnO NRs

The typical aqueous growth solution containing zinc nitrate hexahydrate (Zn(NO_3_)_2_·6H_2_O, 98.0%, Sigma Aldrich) and hexamethylenetetramine ((CH_2_)_6_N_4_, 99.6%, Lachner) at equimolar concentrations (0.05 M) was preheated for 2 h at 93 °C in a closed container. 0 mL, 0.50 and 1 mL polyethyleneimine (PEI, branched, average *M*_w_ ∼ 800 by LS, Aldrich) were added to the solution before the preheating process so as to observe its effect, respectively. The solution color turning yellow was observed upon PEI addition. The pH value of the growth solution was measured to be about 7.1. The ZnO seeded ITO substrates with conductive surfaces turned upside down were immediately immersed into the hot growth solution. Subsequently, the containers were heated at 93 °C for 6 h. ZnO NRs were achieved after rigorously rinsing with deionized water and drying in an oven at 60 °C. Based on the PEI amount characterizing each of the samples prepared at 0, 0.50 and 1 mL PEI, we named the samples ZnO–P0. ZnO–P0.5 and ZnO–P1, respectively.

### 2.3 Characterization

The crystallographic features of the ZnO NR thin films were examined using an X-ray diffractometer (XRD, Rigaku Miniflex 600) equipped with a Co-Kα irradiation source (*λ* = 0.17902 nm) in the range of 2*θ* angles between 20° and 80°. The diffractometer was operated at 40 kV, 100 mA, and a step size of 0.05°. Scanning electron microscope (SEM, Nova NanoSEM 450) analysis was employed to characterize the surface morphology of the top and cross-sectional views of the grown NRs. The microstructure of the prepared sample was monitored by transmission electron microscopy (TEM) using a JEM-2100Plus (Jeol, Tokyo, Japan). The optical properties of the films were analysed with a 150 mm InGaAs integrating sphere module of a UV-Vis-NIR absorption spectrometer (Lambda 1050 PerkinElmer). Furthermore, electrochemical measurements of the produced photoanodes were performed and their interfacial properties were studied using an electrochemical workstation (SP-200 Potentiostat, BioLogic).

### 2.4 Photoelectrochemical and electrochemical measurements

All photoelectrochemical and electrochemical activity, stability and surface area measurements were conducted using a plastic cuvette with a standard three-electrode setup which is constituted by the fabricated samples as the working electrode, Ag/AgCl (saturated KCl) as the reference electrode, and a Pt wire as the counter electrode. 0.5 M of Na_2_SO_4_ aqueous solution with a pH of ∼7 was employed as the electrolyte and purged with nitrogen for 10 min to remove dissolved gases prior to the measurements. The surface area of the photoanode was estimated to be 0.32 cm^2^ and its bare side was attached to the electric connection legs outside of the electrolyte. Unless otherwise stated, the ZnO NRs were front-illuminated with a UV light emitting diode (LED, Roithner LaserTechnik) at 365 nm in all PEC measurements. A net light intensity of ∼3 mW cm^−2^ on the surface of the nanostructured ZnO films was measured with an optical power meter (Opsytec radiometer RM-12). All the electrode potentials measured against the Ag/AgCl reference electrode were converted to the normal hydrogen electrode (NHE) scale by using the equation *E*_NHE_ = *E*_Ag/AgCl_ + 0.2 V.

The *I*–*V* curves for assessing photoelectrochemical performance of the photoanode samples were recorded by linear sweep voltammetry (LSV) in the potential range from 0.2 V to 1.2 V *versus* NHE at a scan rate of 20 mV s^−1^. Chronoamperometry was conducted for the evaluation of the photocurrent response in a light on–off regime at a bias voltage of 0.2 *vs.* NHE. Open circuit potential (OCP) of the electrodes was measured under dark and illumination conditions, and a 0.1 V window centred at OCP was utilized as the potential range in cyclic voltammetry measurements, which were performed in the dark at multiple scan rates within the non-faradaic potential window to estimate the electrochemical active surface area. In electrochemical impedance spectroscopy (EIS) measurements, the impedance spectra were obtained at 10 mV amplitude of the AC signal over a frequency range of 100 kHz to 0.1 Hz. The Mott–Schottky (M–S) analysis was performed with 20 potential steps in the dark at 1 kHz.

The incident photocurrent conversion efficiency (IPCE) and the applied bias photon-to-current efficiency (ABPE) measurements are essential for determining the limiting factors of the photoelectrode performance. In IPCE measurements, monochromatic LEDs (Roithner LaserTechnik) were used as light sources and the efficiency was calculated using1
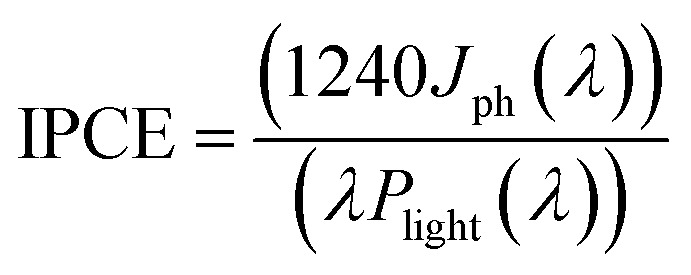
where *J*_ph_ is the photocurrent density (mA cm^−2^), *λ* is the incident light wavelength (nm), and *P*_light_ (mW cm^−2^) is the intensity of the light source at each wavelength. The light intensity of LEDs emitting at 365, 390, and 400 nm (close to band edge absorption of ZnO) was measured to be ∼3, 0.8 and 0.3 mW cm^−2^, respectively. ABPE analogous to the STH (solar-to-hydrogen) efficiency with no bias was also calculated using2
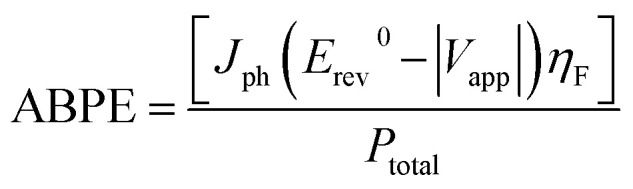
where *V*_app_ is the applied bias between the WE and CE in the two-electrode configuration, *J*_ph_ is the photocurrent density at *V*_app_, *E*^0^_rev_ is the standard state reversible potential for water splitting (1.23 V), *η*_F_ is the faradaic efficiency for hydrogen evolution (*η*_F_ = 1 in this case), and *P*_total_ is the intensity of the light source. The applied bias *V*_app_ of the two electrode set-up is measured with respect to Pt. For this reason, we estimate that the calculation of ABPE represents the overpotential losses originating from the photoanode as well as the activation overpotential developed at the Pt electrode whereas the losses related to ionic resistance of the electrolyte are not taken into consideration.

The photooxidation of Mn^2+^ was also carried out in the three-electrode configuration in 0.5 M Na_2_SO_4_ with 0.01 M MnSO_4_ under back illumination for 3 min with the same light source (LED emitting at 365 nm). The deposition was achieved by passing ∼0.03 C cm^−2^ at 0.7 V *vs.* NHE.

## 3. Results and discussion

**Fig. 1:**
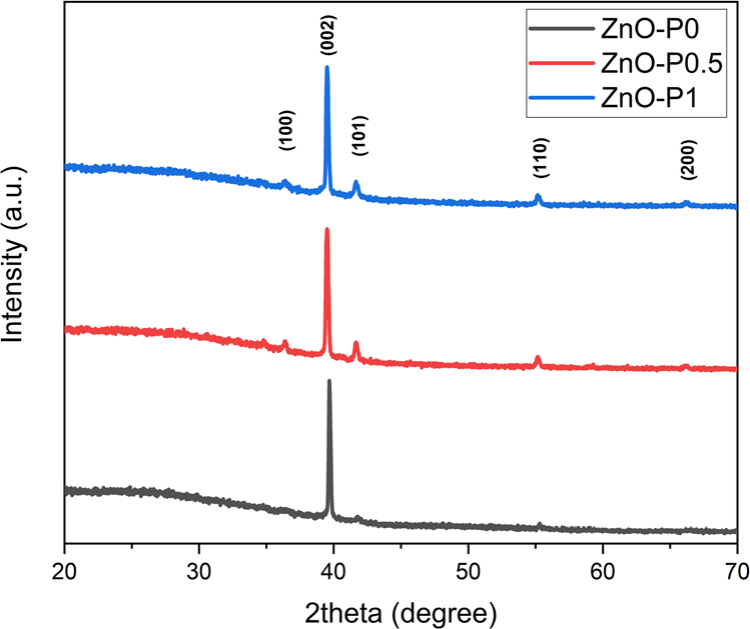
XRD patterns of ZnO–P0, ZnO–P0.5 and ZnO–P1 NR photoanodes.

Fig. 1 shows the XRD patterns of ZnO NRs grown with different PEI quantities as well as the as-prepared ZnO NRs. The presence of sharp and narrow peaks for all the samples provides evidence for good crystallinity quality. The peak at 39.50° accounting for the (002) plane is the most intense peak in the XRD patterns. This peak suggests that ZnO NRs are preferentially oriented along the [001] direction corresponding to a growth perpendicular to the substrate surface. The other weak peaks at 36.40°, 41.67°, 55.13° and 66.24° become more visible in the XRD patterns of the samples grown in the solutions with 0.5 and 1 mL PEI can be indexed to the (100), (101), (110) and (200) planes of the wurtzite crystal structure of ZnO, and no impurity phase was observed.^17^ Such a slight difference was attributed to the polycrystalline layer beneath the ZnO NRs, which was formed at the beginning of the growth process because of the different supersaturation levels and the selective absorption of PEI as its quantity varies.^18^ Additionally, no significant difference in diffraction peak intensity was detected.

**Fig. 2:**
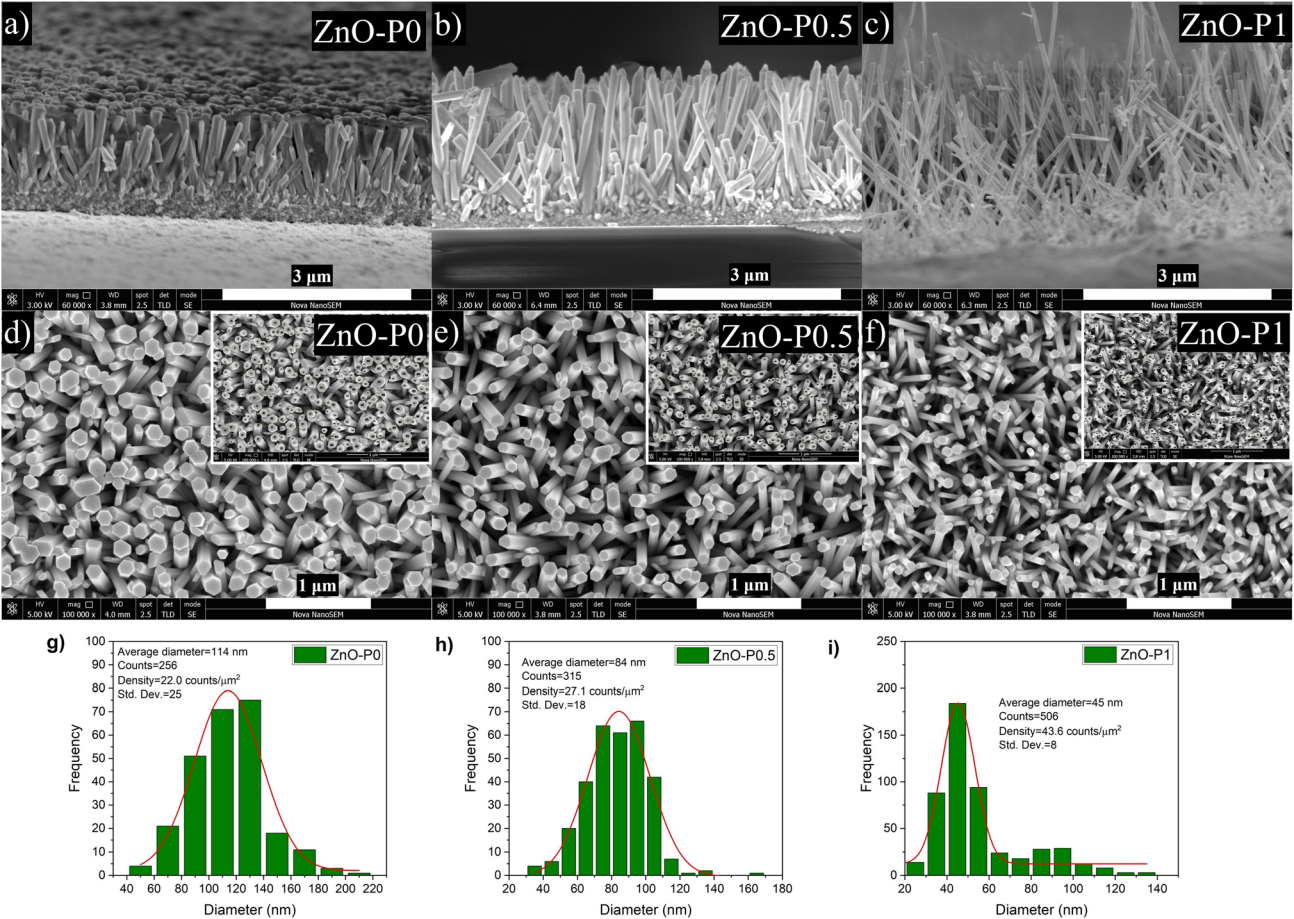
SEM images of ZnO NRs: (a)–(c) cross-sectional, (d)–(f) top-view with insets of diameter distribution, and (g)–(i) diameter distributions.

**Fig. 3:**
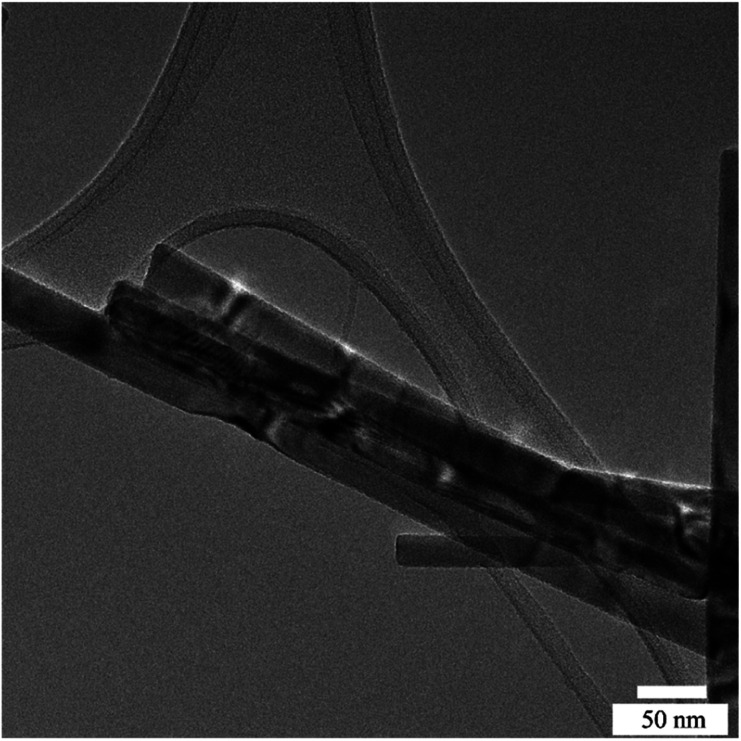
TEM image of ZnO–P1.

Fig. 2a–c indicate the cross-sectional SEM images of the as-prepared ZnO and ZnO NRs with modified morphologies. One can see that ZnO NRs vertically grow on the ITO substrate. As expected from the selective absorption mechanism of PEI, the ZnO nanostructures become thinner and longer with increasing PEI amounts. The measured length of ZnO–P0, ZnO–P0.5 and ZnO–P1 from their cross-sectional area is ∼1144, 1744 and 2155 nm, respectively. The top-view SEM images of the samples shown in Fig. 2d–f also confirm the influence of the PEI amount variation on the ZnO NR growth. In all the cases, the populously untangled NRs with hexagonal shapes form very uniform ZnO arrays. As shown in Fig. 2g–i, the diameter distributions of the ZnO NRs were obtained from the insets of the top-view images (Fig. 2d–f) using Image J software with several plugins. The average particle diameter of ZnO–P0, ZnO–P0.5 and ZnO–P1 was found to be 114 ± 25, 84 ± 18, and 45 ± 8 nm each for few hundreds of NRs, translating to a number density of the NRs of 22.0, 27.1 and 43.6 counts per μm,^2^ respectively. The density exhibits a decreasing trend with an increasing average diameter of NRs. Fig. 3 represents the TEM image of several NRs extracted from the large area arrays of ZnO–P1. The surface morphological analysis shows that each nanorod has clearly visible structural boundaries without any porosity. Addition of 1 mL of PEI to the hydrothermal reaction has decreased the nanorod dimensions along the lateral growth direction and resulted in a spired appearance with a diameter of ∼50 nm. It is shown that TEM observations agree very well with the SEM results.

**Fig. 4:**
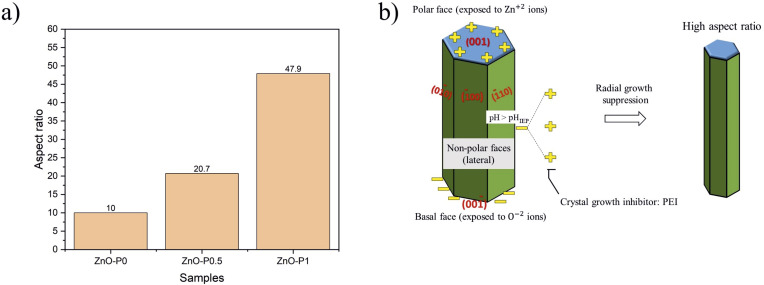
(a) Aspect ratio of the NRs and (b) sketch of the growth mechanism of ZnO NRs *via* PEI.

The variation of the aspect ratio (length over diameter) of the NR samples is given in Fig. 4a. As the PEI concentration increased, ZnO NRs became smaller in diameter and longer in length. For example, introducing a small amount of PEI (0.5 mL) into the reaction mixture led to an almost two times higher aspect ratio. Interestingly, doubling the PEI amount in the reaction yielded a five times greater aspect ratio. Similar results were reported in a study where the aspect ratio of the ZnO NRs increased from 7.9 to 64.4 in the preheated growth solution containing PEI.^19^ In a previous study, the length of ultra-long ZnO arrays fabricated by hydrothermal growth with PEI reached 20–40 μm.^20^

The sketch in Fig. 4b is proposed to identify the growth mechanism of ZnO NRs in the presence of PEI. It is known that hexagonal ZnO crystals have a polar basal O^−2^ plane (001̄), a top positive Zn^2+^ plane (001), and non-polar (1̄00), (1̄10) and (01̄0) lateral facets. The isoelectric pH (pH_IEP_) of ZnO was reported to be 6.4.^21^ Subsequently, the non-polar surfaces are expected to be negatively charged as the above mentioned pH of the growth solution in this study (∼7.1) is greater than pH_IEP_ of ZnO. PEI is a cationic polymer with a vast amount of side amino-groups (–NH_2_). It protonates in a wide range of pH (3–11), and thus tends to be positively charged and attracted to the negatively charged non-polar surfaces of ZnO NRs.^22^ Eventually, PEI inhibits radial crystal growth and yields a high aspect ratio.

An interesting phenomenon was observed on the head-end of ZnO NRs depending on the PEI concentration. In the case of 1 mL PEI addition, the head-ends of ZnO NRs transformed into knob-like structures as shown in Fig. 2f. The strong chelating ability of PEI on Zn^2+^ decreases the probability of free Zn^2+^ ions combining with OH^−^, thus leading to insufficient formation of Zn(OH)^2−^_4_ as the growing units.^23,24^ The excess of OH^−^ attracted to the top polar plane can indeed erode the head-end of the nanorods.

After 6 h of growth, the pH of the growth solution without PEI decreased from 7.08 to 6.63. As for growth solutions with PEI, pH of solution including 0.5 mL PEI decreased from 7.30 to 7.11 while the pH of solution including 1 mL PEI increased from 7.88 to 8.85. These results suggest that a high PEI concentration reasonably raises the pH of the growth solution due to the more emergent OH^−^ ions, leading to prominent erosion. In a report, it has been shown that the deposited ZnO seeds did not turn into rods, but etched from the substrate at high PEI concentrations and PEI caused color change due to the Mannich reaction.^15^ Similarly, we have also observed a color change to darker shades of yellow with increasing PEI amounts, as shown in Fig. S1.† Additionally, a hydrothermal solution containing relatively higher PEI content was also prepared to better explore its effect on NR morphology. Fig. 2Sa† depicts that ZnO precipitation was very poorly formed when 2 mL PEI was introduced. The pH of this solution increased from 8.80 to 9.50 after the growth process, implying the presence of a large amount of OH^−^ ions. The SEM image in Fig. 2Sb† displays the disorderly aligned ZnO NRs with needle tips rather than hexagonal tips as a result of the extreme PEI content. The final shape of the ZnO NRs is apparently determined by the competition between ZnO nucleation and chemical erosion.^16^ Beside the above disadvantage, the calculation of vital surface geometric parameters (in Table 1) would be invalid when the structural geometry deviates from the hexagonal shape. Therefore, 1 mL of PEI amount was chosen as a threshold value in this study to prevent nanorods from more intense surface erosion.

**Table 1:** Geometric features calculated from SEM images (diameter distributions)

Photoanode	Occupied volume (μm^3^)	Free volume%	Total surface area (μm^2^)	Polar surface area (μm^2^)	Non-polar surface area (μm^2^)	Relative ECSA values
ZnO	0.283	75.24	10.43	0.24	9.93	1
ZnO–P0.5	0.289	83.44	14.09	0.16	13.76	1.62
ZnO–P1	0.164	92.35	14.79	0.07	14.64	5.96

Several geometric features of ZnO NRs with particular emphasis on polar and non-polar surfaces are presented in Table 1 in order to deeply investigate the dependence of PEC activity on morphology and certain crystal faces. The volume occupied by NRs was estimated using their average diameter, length and number density deducted from the above statistical distributions (Fig. 2g–i). Accordingly, the free volume% or the void space between NRs was calculated by dividing the volume occupied by NRs by total volume per unit area (1 μm^2^). As ZnO NRs evolve from large diameters to small diameters, the parameter systematically increases. In other words, ZnO NRs prepared with PEI have considerably lower surface coverage. The increased void space can eliminate the restrictions at the semiconductor and electrolyte interface and offer a large number of catalytic sites, which can effectively improve the photoelectrochemical performance.^25^ The total surface area of the NRs involving both polar and non-polar sidewalls was also adequately increased for the small diameter and long NRs. Besides this, polar surfaces account for a very small fraction of the total surface area. The impact of each geometric property on the PEC efficiency of the produced ZnO nanostructures will be further discussed in the following sections.

A crucial factor in evaluating the reactivity of a porous catalytic material is the surface area accessible for the electrolyte solution. Electrochemical active surface area (ECSA) for each sample was estimated from the electrochemical double layer capacitance.^26,27^ A common method for measuring the double-layer capacitance is to record the cyclic voltammograms (CVs) at various scan rates within the potential range where a non-faradaic process occurs. This region is typically a 0.1 V potential window around the open circuit potential (OCP) with the assumption that the measured current in this region is only due to double layer charging.^28^ The OCP measurements (dark/light) for all ZnO NR films are shown in Fig. S3a.† The ΔOCP (the shift in the Fermi level under illumination) refers to the amount of band bending in the semiconductors.^29^ The measured OCP under dark conditions ranges from 0.28 to 0.32 V (*vs.* NHE). Therefore, the potential range for CV measurements was determined to be between ∼0.25 and 0.35 V (*vs.* NHE) with respect to the respective OCP values. In this non-faradaic potential scan range, examples of CV analyses for a variety of scan rates are shown in Fig. S3b–d†.

**Fig. 5:**
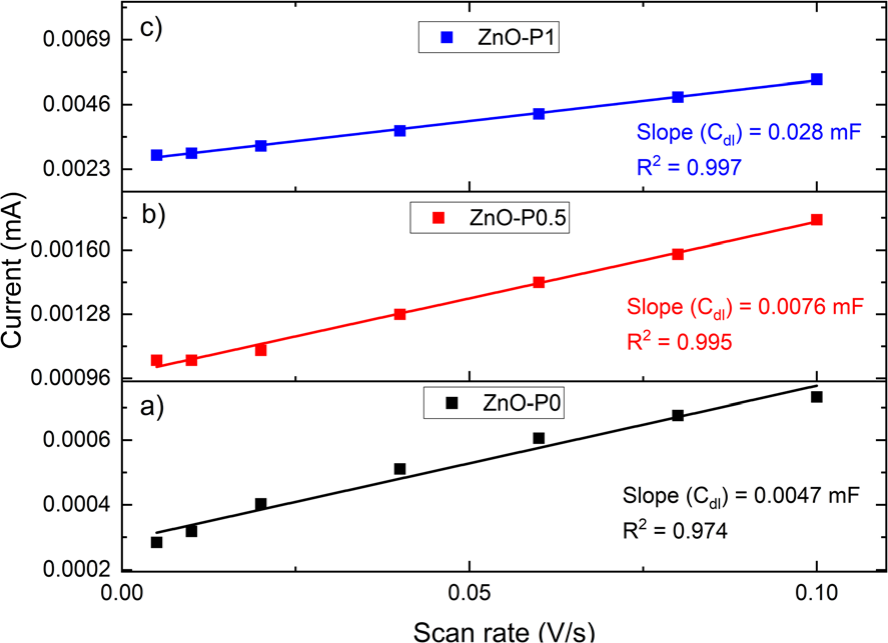
Current-scan rate plots of (a) ZnO–P0, (b) ZnO–P0.5 and (c) ZnO–P1 for determining the electrochemical active surface area.

The electrochemical double-layer capacitance (*C*_DL_) is related to the double-layer current (*i*_C_) and the scan rate (*ν*) by *i*_C_ = *νC*_DL_.^30^ The measured *i*_C_ at 0.35 V (*vs.* NHE) as a function of *ν* has a linear relationship with a slope equal to *C*_DL_, as shown in Fig. 5.The ECSA can be calculated from the extracted *C*_DL_ and the specific capacitance (*C*_s_) of any examined catalyst according to ECSA = *C*_DL_/*C*_S_.^31,32^*C*_s_ describes the capacitance of an ideal flat surface of the electrode material.^33^ One of the major obstacles to a precise evaluation of ECSA has been recognized as the determination of *C*_s_. It has been standard practice to obtain ECSA using a single *C*_s_ value since its value is different for different electrodes. Different values between 20 μF cm^−2^ and 80 μF cm^−2^ are reported for *C*_s_ without accounting for the measuring conditions and the material being utilized.^34^ The most common value of 40 μF cm^−2^ is used here. As shown in Table 1, the relative ECSA values for the three films are in the order ZnO–P1 (5.96) > ZnO–P0.5 (1.62) > ZnO–P0 (1), which is in line with the ranking of the calculated aspect ratio. The ECSA of ZnO–P1 is nearly six times that of ZnO–P0. The increased ECSA for the samples with excellent surface characteristics is beneficial for the increased photoelectrochemical performance.

**Fig. 6:**
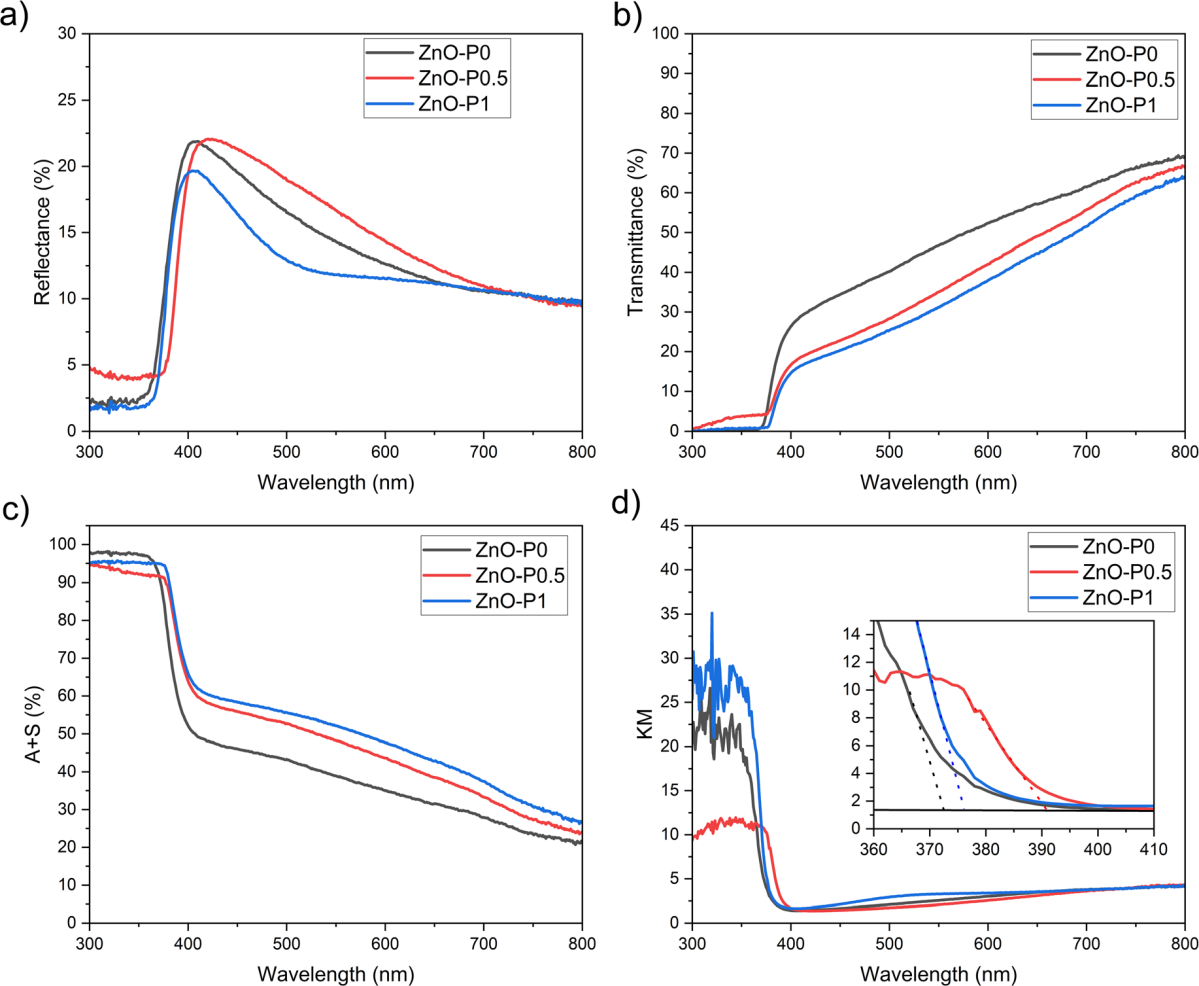
Optical characteristics of ZnO–P0, ZnO–P0.5 and ZnO–P1: (a) reflectance (diffuse and specular), (b) transmittance, (c) absorption plus scattering (*A* + *S* = 100 − *R* − *T*), and (d) KM plots.

The optical response of the ZnO NR thin films was studied in the wavelength range of 300–800 nm. The most salient difference in the reflectance spectra in Fig. 6a and the transmittance spectra in Fig. 6b of the three nanostructures occurs in the visible region and can be explained using scattering events between morphologically varying ZnO NRs. The orientation of small diameter ZnO NR arrays deviates strongly from being vertical to the ITO surface (Fig. 2). This random spatial orientation may give rise to scattering of the incident light. As illustrated in Fig. 6c, absorption plus scattering values (*A* + *S*, %) were also calculated by subtracting transmittance and reflectance from 100% incident light in order to account for the light transmission originating from reflection and scattering. Most of this response arises from scattering in the ZnO films, which effectively channel a significant fraction of the photons into the substrate, where they may be internally reflected and escape unmeasured.^35^ A similar nonzero baseline was observed for the branched TiO_2_ NRs in spite of evidence for the substrate dependency of the scattered light.^9^ ZnO–P1 resulted in the highest absorption plus scattering value of 51% at *λ* = 550 nm in comparison with 47% for ZnO–P0.5 and 38% for ZnO–P0. This reveals that more incident light is internally reflected between the morphologically complex ZnO NRs prepared with PEI in comparison with the as-prepared one prepared without PEI. Moreover, the signal in the UV region drastically increases when photons with energies excite the band gap of ZnO (*λ* < ∼385 nm).

Kubelka–Munk (KM) transformed data are also provided in Fig. 6d to determine optical band gaps of the semiconducting samples. The absorption edge of ZnO NRs was red-shifted from 3.30 eV for ZnO–P0 to 3.26 eV for ZnO–P1 and 3.16 eV for ZnO–P0.5, which may be due to the quantum size effect as a consequence of the PEI inhibiting ZnO NR lateral growth. This similar observation was also reported by other researchers.^36^

**Fig. 7:**
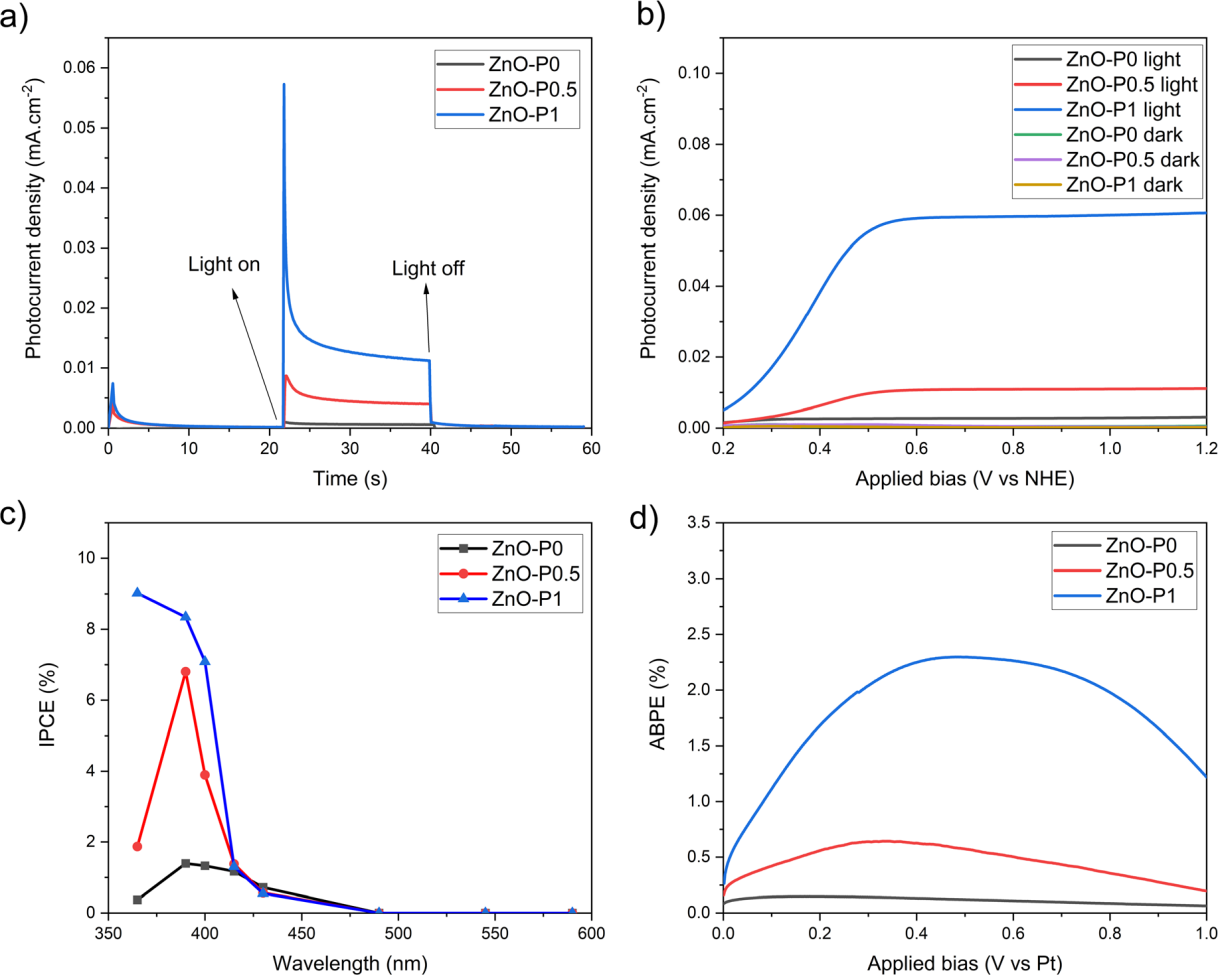
Photoelectrochemical properties of the produced ZnO NRs: (a) chronoamperometric photoresponse at an applied bias of 0.2 V *vs.* NHE, (b) linear sweep voltammogram, (c) IPCE spectra at an applied bias of 0.5 V *vs.* NHE, and (d) ABPE spectra.

The photocurrent transient responses of ZnO–P0, ZnO–P0.5 and ZnO–P1 photoanodes monitored by chronoamperometry with no external bias (0 V *vs.* Ag/AgCl) in a light on–off cycle are shown in Fig. 7a. As soon as the light turns on, a sharp transient decay is obtained for ZnO–P1 whereas less considerable transient decays are observed for ZnO–P0 and ZnO–P0.5. The photocurrent decay is principally attributed to the recombination of holes trapped at the surface states with electrons in the conduction band.^37^ The steady photocurrent behavior indicates that negligible surface recombination emerged in ZnO–P0 and ZnO–P0.5 photoanodes due to fewer surface states and rapid charge separation. In contrast, the severe surface recombination rate generated a significant photocurrent transient decay of the ZnO–P1. ZnO NRs with diameters below 40 nm were found to suffer from surface charge recombination as the number of mid-band-gap surface states increased.^38^ The average NR diameter of ZnO–P1 is around this critical margin, and thus the charge kinetics at the interface can be expected to be affected by the surface traps. It is also worth noting that the highest photocurrent density was achieved with ZnO–P1 (0.012 mA cm^−2^ under 0 V *vs.* Ag/AgCl).

Linear sweep voltammograms (*I*–*V*) were recorded from 0.2 to 1.2 V *vs.* NHE for the as-prepared ZnO NRs and ZnO NRs grown with different PEI quantities in the presence and absence of light, and presented in Fig. 7b. No measurable current was observed in any dark scans. For all photoanodes, the photocurrent increased when bias swept towards more positive values and the saturation current was reached at 0.5 V *vs.* NHE. The produced ZnO NR photoanodes were irradiated with UV light (3 mW cm^−2^), and ZnO–P0, ZnO–P0.5 and ZnO–P1 exhibited photocurrent densities of 0.003, 0.01 and 0.06 mA cm^−2^ at 0.5 V *vs.* NHE, respectively. It was found that the thin nanorods show more efficient PEC performance than the thick nanorods. The highest photocurrent density of ZnO–P1 was attributed to its superior surface properties, *i.e.* the largest electrochemical active surface area and largest void space between NRs, possibly with a vast number of catalytic sites. Such a trend for the photocurrent density variation was previously achieved for ZnO NRs with diameters from 45 nm to 275 nm.^13^ A more detailed comparison of these results with other literature values is presented in Table S1.† Consistently, ZnO NRs with a high aspect ratio mostly exhibit the maximum photocurrent value; however, the analogy between the results is not straightforward due to the uncommon use of electrolyte solution, irradiation source and intensity in PEC experiments. The polar surfaces are purposed to be catalytically more active than the non-polar surfaces of ZnO NRs.^39,40^ As nanorods become small in diameter and grow in length (Fig. 4b), the hexagonal structure loses polar surface area while gaining non-polar surface area (Table 1). Large NR diameters provide prevailing polar surfaces, but limit the total surface area due to the limited lateral surfaces. On the other side, the non-polar fraction is the only significant factor contributing to the total surface area when decreasing the average NR diameter. Therefore, in our case, this opposing behaviour may explain the PEC efficiency order of the fabricated photoelectrodes.

IPCE and ABPE tests are crucial for evaluating the photoelectrode performance. The IPCE spectra recorded at 0.5 V *vs.* NHE (0.3 V *vs.* Ag/AgCl) and shown in Fig. 7c display the spectral photoresponse generating the total PEC performance of each electrode. As highly expected from the ZnO material, IPCE values for all the semiconducting specimens culminate around 385 nm, corresponding to its band gap, and severely disappear at around 400 nm. The unique effect of PEI is evident in that ZnO NRs produced with it exhibit higher IPCE values with respect to the as-prepared ZnO NRs. IPCE is influenced by the efficiencies of the three fundamental processes concerned in the PEC reaction: charge generation (*η*_e^−^/h^+^_), charge transport within the electrode (*η*_transport_), and charge transfer at the electrode/electrolyte interface. The charge generation efficiency is very relevant to the light absorption capacity.^41^ The optical absorption edges of ZnO–P1 and ZnO–P0.5 can be activated with slightly less energetic photons compared to ZnO–P0 (Fig. 6d), yet the three nanosystems have very similar light absorption below 400 nm (Fig. 6c); thus absorption alone cannot fully explain the performance ranking between the three ZnO NR photoanodes.

When it comes to charge transport (collection) efficiency, one unique aspect of high-aspect-ratio NRs is being long in the direction of incident light for maximum light absorption, while at the same time being thin in the direction of carrier collection to improve efficient extraction of the photogenerated carriers.^42^ Since electron–hole pairs formed in the vicinity of the depletion region are rapidly separated by the built-in field, the collection probability is quite high for a hole generated within a diffusion length, making it less susceptible to recombination.^43,44^ On this basis, ZnO NRs with smaller diameters can effectively transport holes for water oxidation at the interface. In the following section, we provide visual evidence for more favourable hole extraction efficiency within tiny ZnO NRs using a hole imaging agent. The charge transfer efficiency is another component affecting IPCE, and it is primarily related to the total surface area at semiconductor and liquid interfaces. It has emerged that the total surface area as well as electrochemical active surface area of ZnO NRs substantially increases with increasing PEI contribution (Table 1), yielding a high IPCE value.

A similar ranking was observed for the ABPE spectra measured in a two electrode configuration so as to evaluate the photoresponse of the samples as a function of the applied bias.

As demonstrated in Fig. 7d, the maximum ABPE for ZnO–P0, ZnO–P0.5 and ZnO–P1 is 0.15% (at 0.2 V *vs.* Pt), 0.65% (at 0.35 V *vs.* Pt) and 2.30% (at 0.5 V *vs.* Pt), respectively. These conversion efficiency values are also consistent with the previously reported values for pure ZnO NRs under 365 nm UV irradiation.^12^ Electrochemical water separation requires a voltage of 1.23 in theory, but in practice 1.8 V is required to overcome the activation barrier of the reaction.^45^ Hence, low input voltages are not sufficient to generate enough photocurrent. Consequently, the effective charge transport and transfer in ZnO samples constituted by thinner NRs give rise to IPCE and ABPE values compared to those of the as-prepared ZnO NRs.

**Fig. 8:**
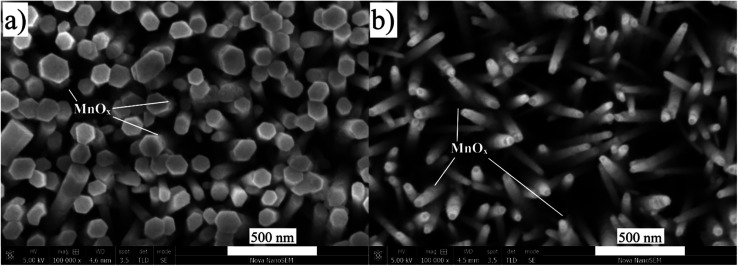
Top view SEM images after photodeposition of Mn^2+^ of (a) ZnO–P0 and (b) ZnO–P1.

Several reports suggest that the hole extraction efficiency may depend on the nanorods' dimensions.^46,47^ A photocatalytic system with a proper structure can shorten the transport path for holes to the surface and allow electrolyte to diffuse into thin films as mentioned above. One of the interesting approaches is to use Mn^2+^ as the photogenerated hole imaging agent to demonstrate that a shorter hole diffusion length leads to higher charge separation efficiency.^48,49^ The acquired results provide visual evidence for the hole transport process. Electrooxidation potential of MnO_*x*_ in the dark begins at 0.75 V *vs.* NHE as shown in Fig. S4.† A lower potential than this (0.7 V *vs.* NHE) was applied for the photodeposition of MnO_*x*_ on the surface of ZnO NRs to utilize only photogenerated holes for the oxidation. As shown in Fig. 8a and b, MnO_*x*_ was barely grown on the surface of ZnO–P0 (without a surfactant) while needle-like MnO_*x*_ was uniformly deposited especially on the nanorod lateral surfaces of ZnO–P1. Different MnO_*x*_ deposition sites imply different transport paths for the photogenerated holes as the average diameter of the NRs shrinks with the effect of PEI. The SEM image of MnO_*x*_ photodeposition on ZnO–P0.5 is also given in Fig. S5.† For more quantitative data on the amount of MnO_*x*_ deposited on each ZnO NR sample, EDX analyses are provided in Fig. S6† and the atomic percentage of Mn was found to be 1.61, 2.61 and 3.54 for ZnO–P0, ZnO–P0.5 and ZnO–P1, respectively. These results confirm that the ZnO–P0.5 and ZnO–P1 photoanodes use the space between the thin NRs and benefit from large semiconductor surface and electrolyte interfaces for efficient transfer of photogenerated holes whereas ZnO–P0 fails to take advantage of the voids for improved photocurrent.

**Fig. 9:**
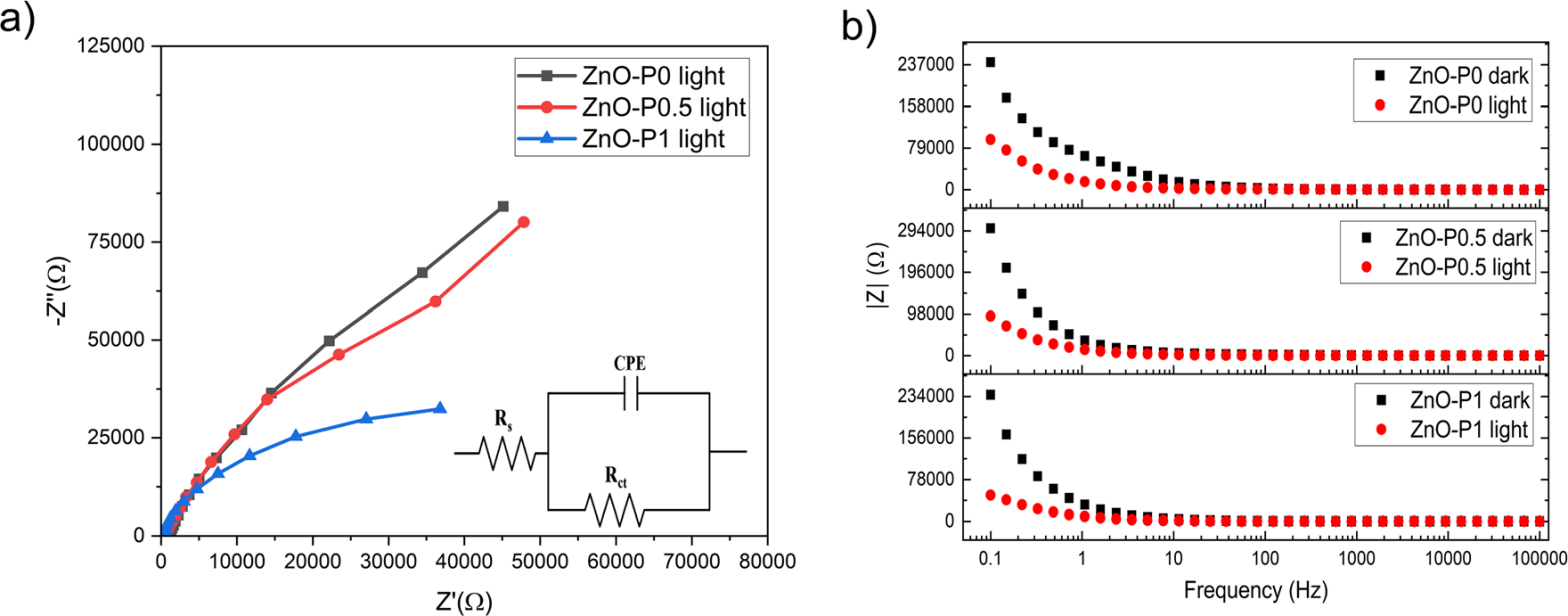
EIS data of the samples, (a) Nyquist plots (points) under illumination with model fits (lines) and (b) Bode plots under dark and illumination. The inset of (a) is an equivalent circuit model.

Electrochemical impedance spectroscopy (EIS) was used to investigate the charge kinetics at the ZnO NR electrodes and electrolyte interface. The Nyquist plots (points) along with their model fits (lines) for three of the morphologically different ZnO NRs under illumination are depicted in Fig. 9a. The obtained curves were fitted into an equivalent circuit (Randle's cell, in the inset) that comprises the resistance of electrolyte solution (*R*_s_), the charge transfer resistance (*R*_ct_), and the constant phase element (CPE), which is more suitable for the inhomogeneity of the photoanode surface. Table 2 shows the calculated values of these proposed electrical elements. *R*_s_ values of all three samples are low and comparable to each other. The lower *R*_ct_ in ZnO–P1 as compared to ZnO–P0.5 and ZnO–P0 suggests the lower electron transport resistance and much more improved interface charge transfer kinetics due to the significantly enhanced electrochemical active surface area, which leads to more interaction between the ZnO surface and electrolyte solution. Similarly, Han *et al.* reported a drastic decrement in *R*_ct_ depending on the morphology of ZnO. It was suggested that the oxygen vacancies on the ZnO flower-rod nanostructure decrease the probability of charge carrier recombination; thus charges move rapidly from ZnO.^50^

**Table 2:** Solid state properties derived from EIS, MS and Bode analyses

Photoanode	*R*_s_ light (Ω)	*R*_ct_ light (kΩ)	*N*_D_ (cm^−3^ × 10^20^)	*V*_fb, pH=7_ dark (V *vs.* NHE)	*V*_fb, pH=7_ dark (V *vs. E*_vacuum_)	*τ*_n_ (s)
ZnO–P0	375	484	0.3	0.077	−4.57	0.03
ZnO–P0.5	274	332	18.9	−0.053	−4.44	0.10
ZnO–P1	216	80	7.1	−0.069	−4.43	0.05

The Bode plots in Fig. 9b are given under both dark and light illumination to understand the light sensitivity of the electrodes. Although the absolute value of the total resistance of each electrode decreased with exposure to light, ZnO–P1 exhibited the lowest total resistivity due to the higher current produced during illumination.

**Fig. 10:**
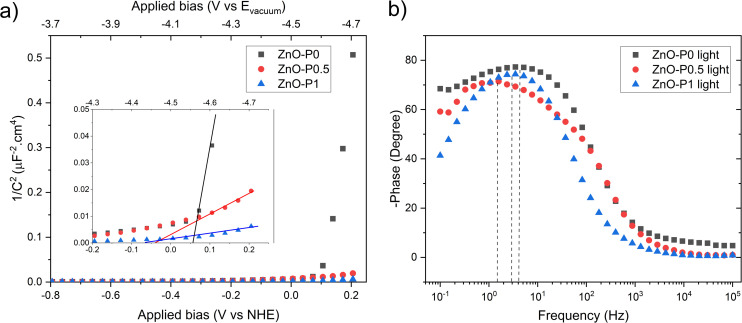
(a) Mott–Schottky plots with an expanded view of the intercepts and (b) Bode phase plots.

Mott Schottky (MS) analysis was performed to estimate flat band potential (*V*_fb_) and the donor density (*N*_D_) of the samples, as shown in Fig. 10a. The calculations are based on the MS equation:3
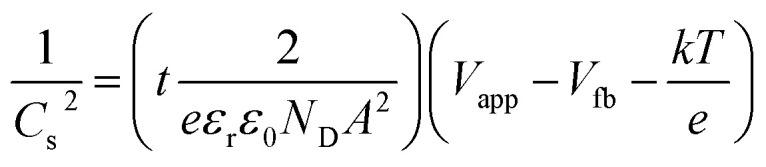


where *C*_s_, *e*, *ε*_r_, *ε*_0_, *N*_D_, *A*, *V*_app_, *V*_fb_, *k* and *T* are the space charge capacitance, the elementary electric charge, the dielectric constant of ZnO, the vacuum permittivity, the carrier density, the contact area of the electrode, the applied bias *vs.* NHE, the flat band potential, the Boltzmann constant and the absolute temperature. The gradient and *x*-intercept of the linear part of the MS plot determine *N*_D_ and *V*_fb_, respectively. Table 1 summarizes the obtained *N*_D_ and *V*_fb_ values for all samples. An increase in donor density was observed for ZnO photoanodes prepared with PEI. The shift in the Fermi level resulting from increased donor density causes substantial band bending, which in turn leads to enhancement in the electric field in the space charge region and promotes the charge separation.^51^ As for *V*_fb_, all three electrodes presented values close to that previously found for blank ZnO (−0.13 V *vs.* NHE).^52^ Nevertheless, both the ZnO–P0.5 and ZnO–P1 have slightly shifted values towards more negative potential (*vs.* NHE or closer to the vacuum level) compared to ZnO–P0. The more negative shift in flat band potential with respect to water reduction potential promotes the spontaneous water splitting process.^53,54^ Moreover, the free electron lifetimes (*τ*_*n*_) of the illuminated electrodes can be determined from *τ* = 1/2π*f*_peak_, where *f*_peak_ is the peak frequency at the minimum phase angle in the Bode phase plots shown in Fig. 10b. The calculated *τ*_*n*_ values are also summarized in Table 2. The observed electron lifetime is in the order: *τ*_*n*_(ZnO–P0.5) > *τ*_*n*_(ZnO–P1) > *τ*_*n*_(ZnO–P0). The shortest electron lifetime and thus the fastest charge recombination rate originated from ZnO–P0. The tailored morphology contributes to the charge separation and then enhances the photoelectrochemical activity.

In photoelectrochemistry, the potentials are usually given with respect to NHE (*E*^0^_NHE_ (H^+^/H_2_) = 0 V) whereas solid state properties such as flat band potential are referenced to the absolute vacuum in the field of physics.^55^ The NHE is reported to lie at −4.5 eV (at 298.15 K) with respect to the vacuum level on the energy scale.^56^ The negative sign here comes from that the energy in the physical scale *vs.* the vacuum level moves towards negative values while the absolute potential moves towards positive values: *E*_(abs)_/[V] = −*E*_(AVS)_/[eV].^57^ For comparative purposes, we provide the absolute vacuum energy scale on the top axis of the MS plots (Fig. 10a). Furthermore, the protonation and deprotonation of the hydroxylated ZnO surface in contact with aqueous electrolyte induce positive and negative net charges at the surface, respectively. Accordingly, *V*_fb_ becomes a pH-dependent quantity due to this charge imbalance at the surface. For this reason, the obtained *V*_fb_ values of the photoanode were also given *vs.* the vacuum level at the pH value of the electrolyte solution used in this work (Table 2).

**Fig. 11:**
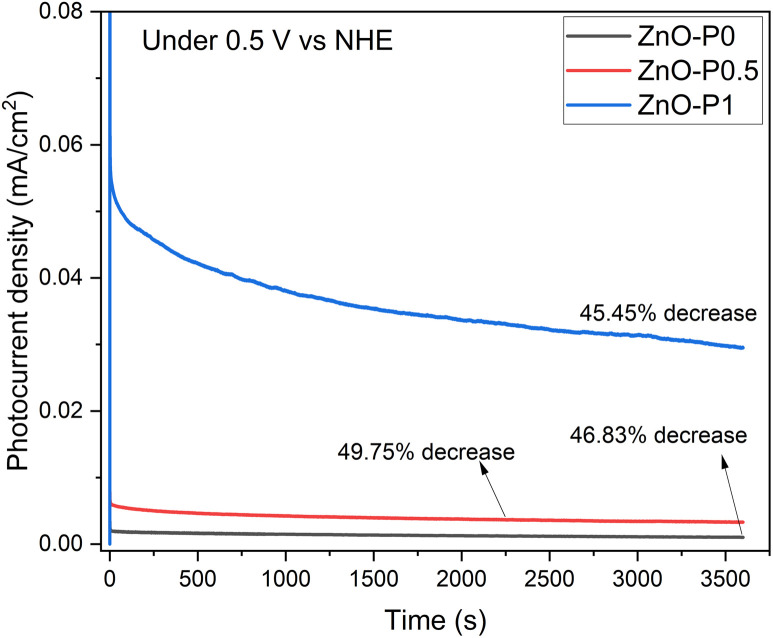
Stability test of photoelectrodes for 1 h under illumination at 0.5 V *vs.* NHE.

The long term stability of the fabricated electrodes was tested by chronoamperometric measurements under illumination at 0.5 V *vs.* NHE in 0.5 M Na_2_SO_4_ electrolyte, as shown in Fig. 11. The photocurrent density of the three samples significantly decreased in the first 20 minutes, and after an hour, this drop rate reached about 45–50%. This result demonstrated that ZnO NRs are prone to corrosion without a proper design of the protection layer.

It should be mentioned again that the photocurrent efficiency is governed by surface chemical reactions (charge utilization) as well as charge generation and charge separation/transport. Surface trap states are prevalent for small diameter (<40 nm) ZnO nanorods.^38^ Since the average diameter of ZnO–P1 is ∼45 nm, some of the components of its size distribution below the critical value of 40 nm can cause surface recombination. In fact, surface recombination has a particularly detrimental impact on the photoresponse of an electrode. However, the large surface area of small nanorods potentially offers kinetically more favorable conditions for redox reactions to occur.^58^ As we mentioned in the discussion of the EIS results, the charge transfer resistance was found to increase with decreasing surface area, indicating that there is more space among NRs for surface redox reactions. Apart from this, the prolonged electron life times of the PEI-assisted grown ZnO NRs with high surface areas support better charge transport compared to ZnO NRs grown without PEI. Overall, we contemplate that the increased surface area of ZnO–P1 compensates for the ohmic loss associated with the surface recombination.

Based on the above discussions, the efficient PEC performance of the ZnO NRs prepared with PEI as a surfactant can be elucidated using the following aspects. First, the large aspect ratio and void space between NRs resulted in faster charge transfer kinetics at the ZnO NRs/electrolyte interface, which was confirmed by EIS data. The second argument is that ZnO NRs with small diameters facilitate hole transport for water oxidation, augmenting charge separation. The third consideration is the higher light absorption capacity associated with the quantum size confinement, indicated as band gap manipulation in the KM plots. Additionally, the scattering phenomena due to the internal reflection between smaller nanosized NRs have potential to increase light harvesting capacity in the visible region.

## 4. Conclusion

The model nanoforest structure of ZnO NRs with three different exemplary morphologies was utilized to examine the relationship between surface properties and electrochemical/photoelectrochemical properties. The geometric features of ZnO NRs grown with the hydrothermal method were progressively modified by varying the PEI amount (0, 0.5 and 1 mL), which inhibited the lateral growth of ZnO crystals. A comprehensive analysis of SEM images enabled the estimation of the crucial surface characteristics of NRs. The aspect ratio and void space of ZnO NRs proportionally increased with increasing PEI concentration which correlates with the increased specific surface area and hence also with an increase in the electrochemically available area. The obtained nanostructures with small diameters of ∼45 nm demonstrated very effective PEC water splitting performance. This high PEC activity is attributed to its enhanced electrochemical surface area for more interaction at the ZnO/electrolyte interface (interfacial charge kinetics) and enhanced charge collection/separation coming from a shorter photogenerated hole diffusion distance, leading to a more favorable oxygen evolution reaction. Additionally, the scattering effect from internal reflections between randomly oriented tiny NRs in the visible range may be a contributing factor to the overall PEC efficiency. We expect that the present study offers a profound understanding of the connections between photoelectrochemical characteristics and nanoarchitecture along with the detailed mechanism of nanocrystal growth.

## Data availability

The data that support the findings of this study are available from the corresponding author upon reasonable request.

## Conflicts of interest

The authors declare no conflict of interest.

## Supplementary Material

NA-005-D3NA00089C-s001
