# Supplementary material for: Comprehensive evaluation of photoelectrochemical performance dependence on geometric features of ZnO nanorod electrodes
Source: Nanoscale Adv. 2023 May 24;5(11):3091–103. doi: 10.1039/d3na00089c (PMC10228492; doi:10.1039/d3na00089c)
Supplement: NA-005-D3NA00089C-s001 [file NA-005-D3NA00089C-s001.pdf]

Electronic Supplementary Material (ESI) for Nanoscale Advances.
This journal is © The Royal Society of Chemistry 2023

Comprehensive evaluation of photoelectrochemical performance dependence on geometric feature of ZnO nanorod electrodes

Ali Can Guler[a], Jan Antos[a], Milan Masar[a], Michal Urbanek[a], Michal Machovsky[a] and Ivo Kuritka*[,a,b]

[a]Centre of Polymer Systems, Tomas Bata University in Zlin, Tr. T. Bati 5678, 760 01 Zlin, Czech Republic

[b]Department of Chemistry, Faculty of Technology, Tomas Bata University in Zlín, Vavrečkova 5669, 760 01 Zlín, Czech Republic

Email addresses: guler@utb.cz; antos@utb.cz; masar@utb.cz; murbanek@utb.cz; machovsky@utb.cz; *corresponding author: kuritka@utb.cz

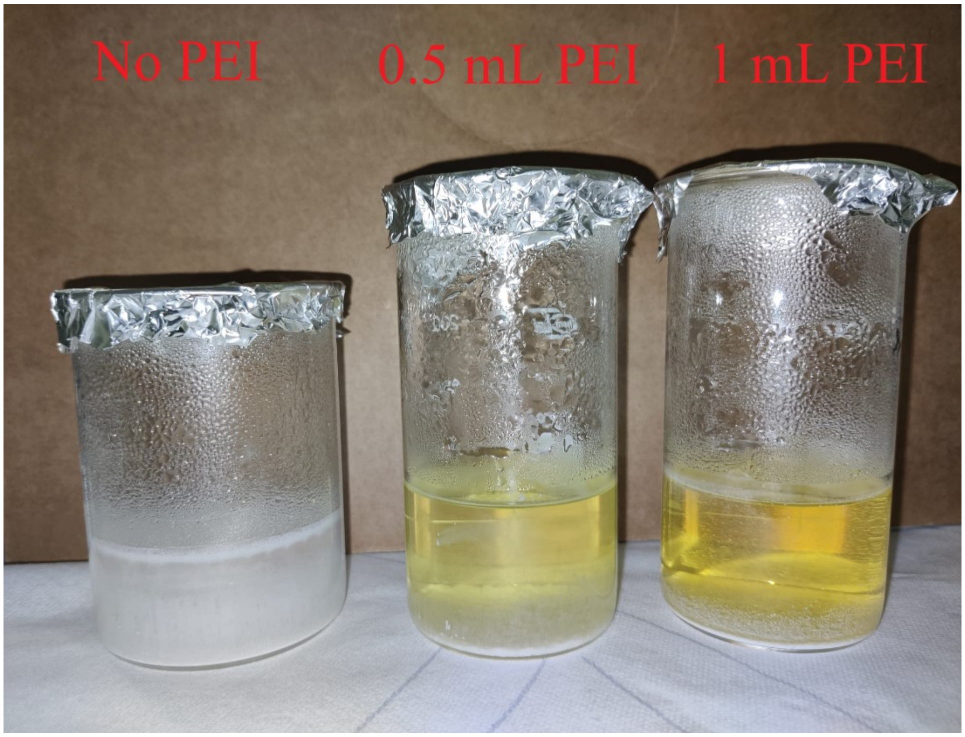

Fig. S1 Color change of hydrothermal growth solution depending on amount of PEI surfactant.

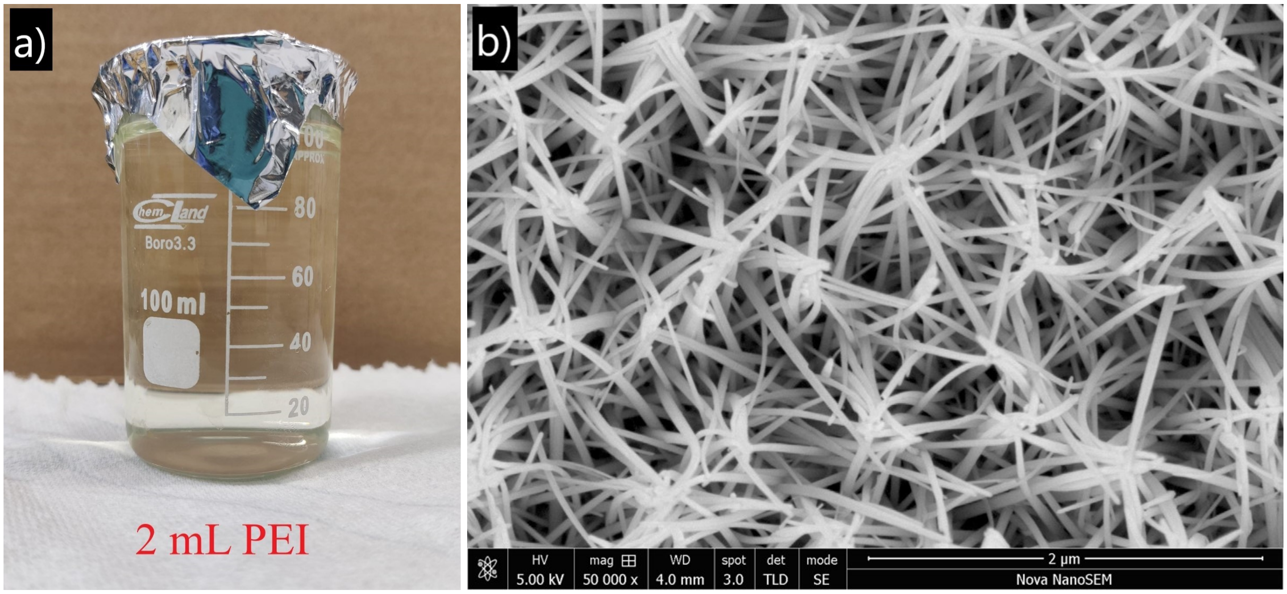

Fig. S2 a) Hydrothermal growth solution containing 2 mL PEI and b) Corresponding SEM images of ZnO NRs prepared via hydrothermal solution containing 2 mL PEI.

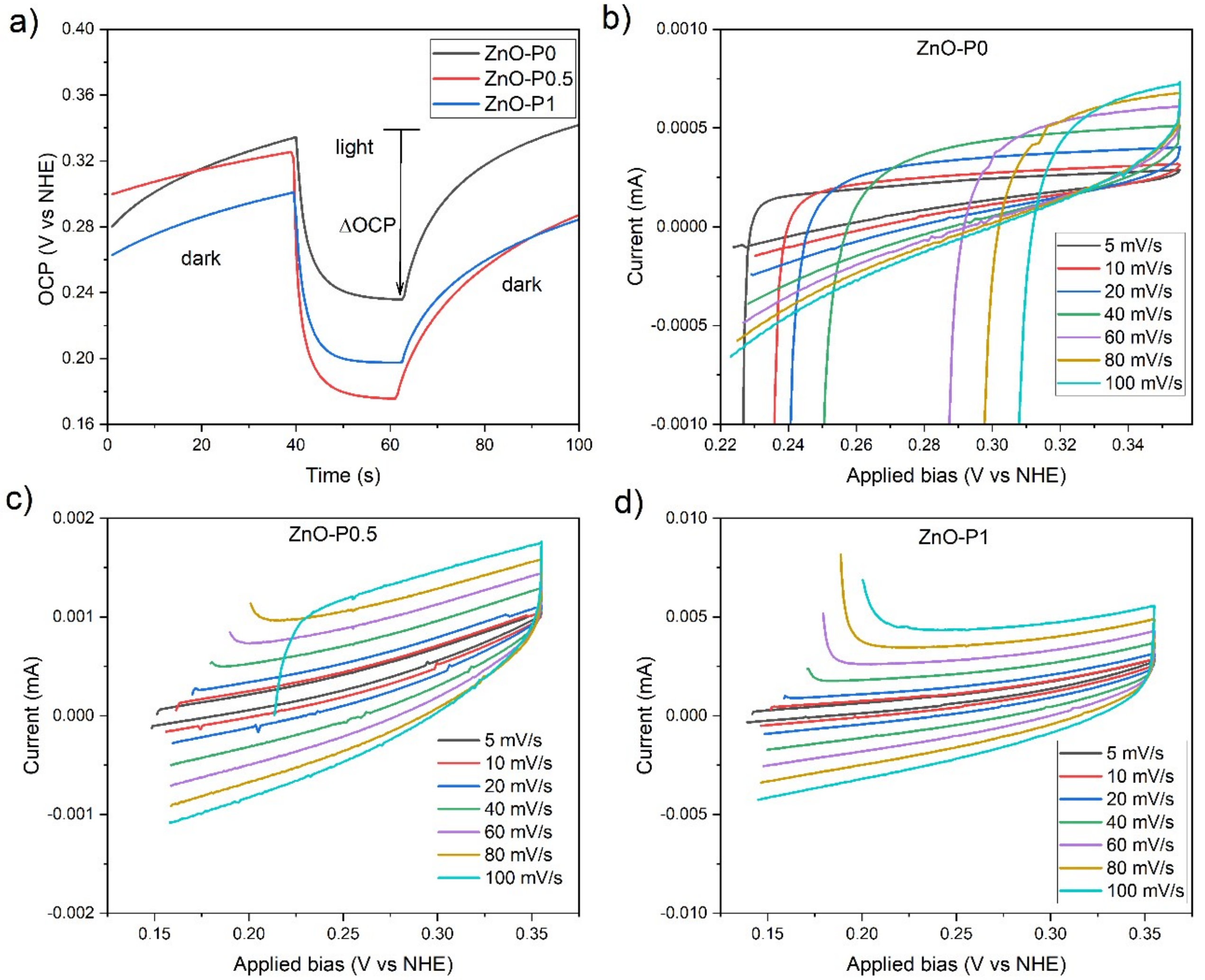

Fig. S3 a) Open circuit potential measurements in the dark and light for the all samples. Cyclic voltammograms at various scan rates of b) ZnO-P0, c) ZnO-P0.5 and d) ZnO-P1.

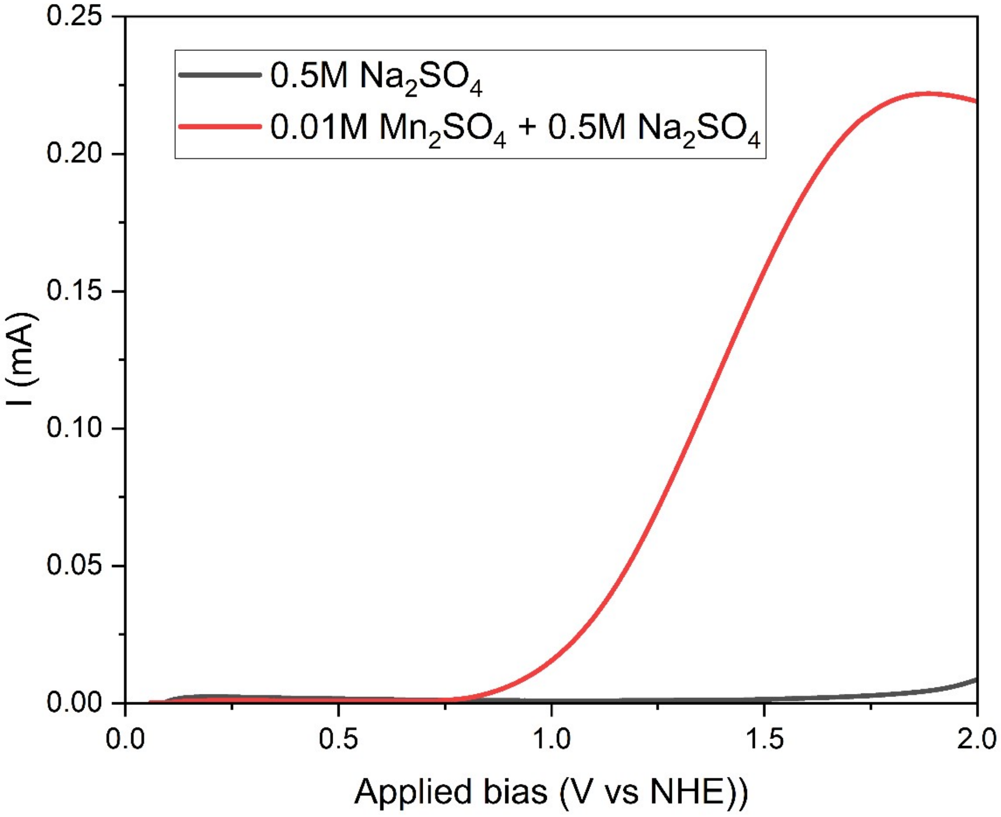

Fig. S4 Dark current of ZnO NRs electrode in 0.5 M Na_2SO_4 and 0.5 M Na_2SO_4 together with 0.01 M $MnSO_4$.

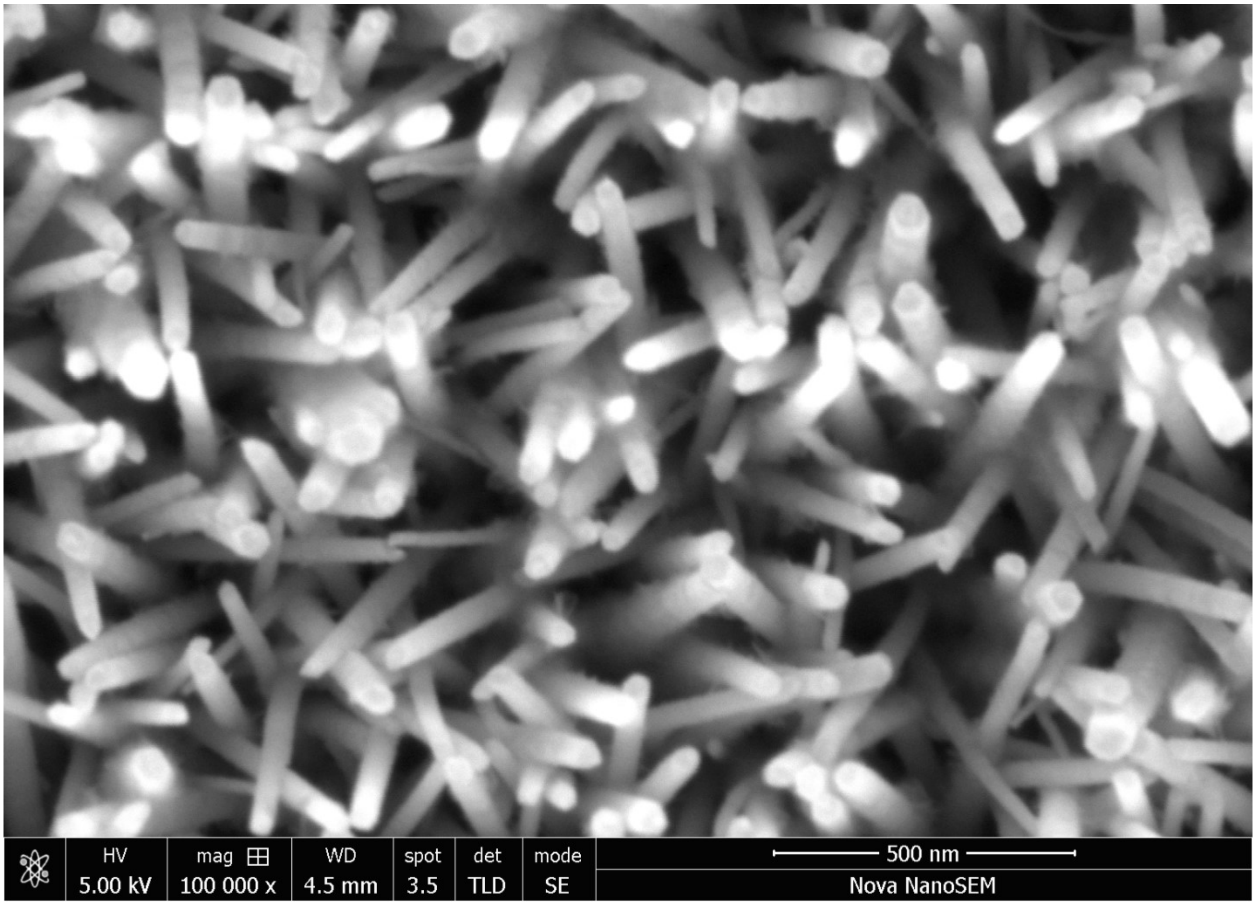

Fig. S5 Top view SEM image of ZnO-P0.5 after photodeposition of Mn^{+2}.

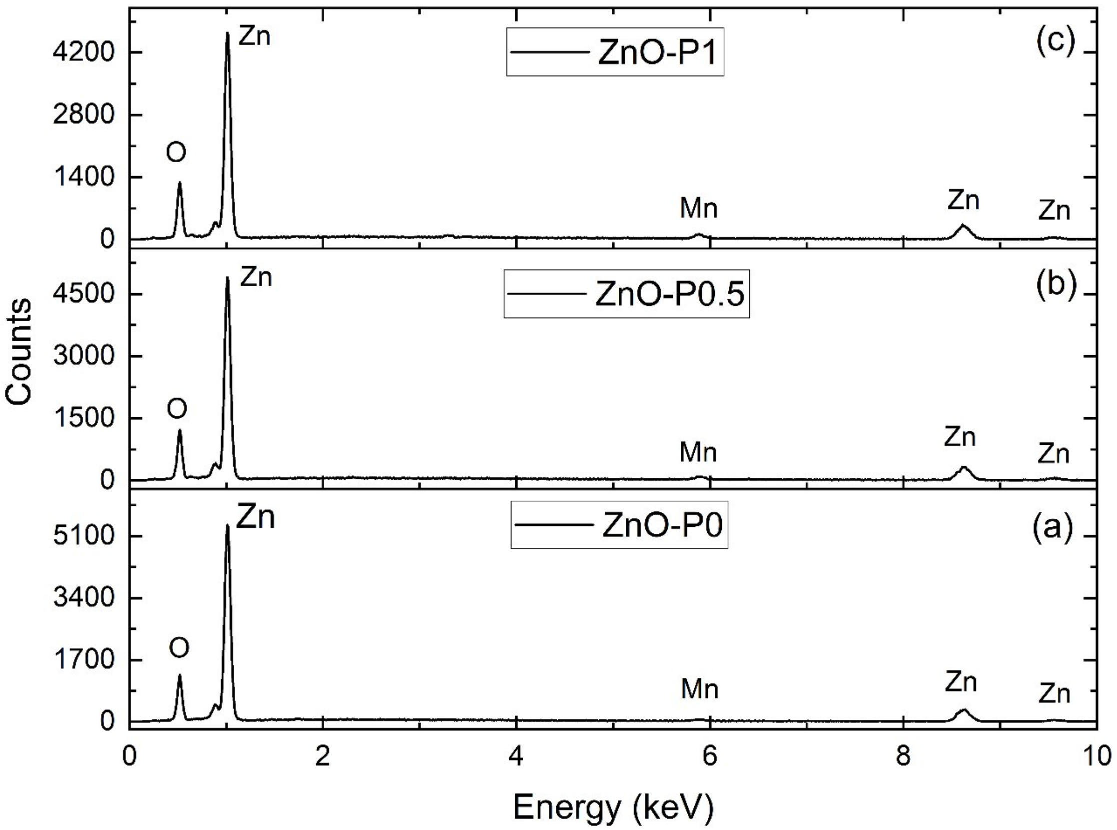

Fig. S6 EDX spectrum of (a) ZnO-P0, (b) ZnO-P0.5 and (c) ZnO-P1 after photodeposition of Mn^{+2}.

Table S1. Comparative study for the PEC performances of ZnO NRs under various experimental conditions.

Average Diameter (nm)	Synthesis method	Light source and intensity	Electrolyte solution	Photocurrent density (mA/cm^2)
45 [1]	Hydrothermal	UV LED (365 nm) with 0.4 mW/cm^2	No data	0.35 at 0.3 V vs SCE
69 [2]	Hydrothermal	halogen lamp with 100 mW/cm^2	0.1 M Na_2S + 0.1 M Na_2SO_3	0.48 at 0.5 V vs Ag/AgCl
120 [3]	Hydrothermal	UV LED (365 nm) with 11.5 mW/cm^2	0.1 M NaOH	2.25 at 1 V vs RHE
No data [4]	RF sputtering	150 W tungsten – halogen lamp with 125 mW/cm^2	0.5 M Na_2SO_4	0.40 at 1 V vs Ag/AgCl

400 [5]	Electrodeposition	150 W Xenon lamp with 100 mW/cm^2	NaOH	0.39 at 0.5 V vs SCE
42 [6]	MOCVD	300 W Xenon lamp with 100 mW/cm^2	0.5 M Na_2SO_4	0.27 at 2 V vs RHE
70 [7]	Hydrothermal	Xenon lamp with 75 mW/cm^2	0.5 M Na_2SO_4	0.4 at 1.4 V vs RHE
45 in this study	Hydrothermal	UV LED (365 nm) with 3 mW/cm^2	0.5 M Na_2SO_4	0.06 at 0.5 V vs NHE

References

1 E. S. Babu, S. K. Hong, T. S. Vo, J. R. Jeong and H. K. Cho, *Electron. Mater. Lett.*, 2015, **11**, 65–72.

2 H. J. Tan, Z. Zainal, Z. A. Talib, H. N. Lim, S. Shafie, S. T. Tan, K. B. Tan and N. N. Bahrudin, *Ceram. Int.*, 2021, **47**, 14194–14207.

3 K. Govatsi, A. Seferlis, S. G. Neophytides and S. N. Yannopoulos, *Int. J. Hydrogen Energy*, 2018, **43**, 4866–4879.

4 K. S. Ahn, S. Shet, T. Deutsch, C. S. Jiang, Y. Yan, M. Al-Jassim and J. Turner, *J. Power Sources*, 2008, **176**, 387–392.

5 A. Rokade, S. Rondiya, V. Sharma, M. Prasad, H. Pathan and S. Jadkar, *J. Solid State Electrochem.*, 2017, **21**, 2639–2648.

6 M. A. Hassan, A. Waseem, M. A. Johar, I. V. Bagal, J. S. Ha and S. W. Ryu, *J. Mater. Chem. A*, 2020, **8**, 8300–8312.

7 C. F. Liu, Y. J. Lu and C. C. Hu, *ACS Omega*, 2018, **3**, 3429–3439.
